# Comparative Pharmacokinetics of Hydrophilic Components in *Salvia miltiorrhiza Bge*. and *Carthamus tinctorius L.* in Rats That Underwent Cerebral Ischemia Reperfusion Using an HPLC-DAD Method

**DOI:** 10.3389/fphar.2019.01598

**Published:** 2020-01-24

**Authors:** Xixi Zhao, Li Yu, Yulin Chen, Yu Wang, Haitong Wan, Jiehong Yang

**Affiliations:** ^1^ College of Life Science, Zhejiang Chinese Medical University, Hangzhou, China; ^2^ College of Basic Medical Sciences, Zhejiang Chinese Medical University, Hangzhou, China

**Keywords:** danshensu, hydroxysafflor yellow A, salvianolic acid A, salvianolic acid B, compatibility, middle cerebral artery occlusion, pharmacokinetics

## Abstract

**Background:**

In China, the combination of herb *Salvia miltiorrhiza Bge.* (Danshen) and *Carthamus tinctorius L*. (Honghua) is an effective treatment for stroke. A previous study showed that the combination of four herbal components: danshensu (DSS), hydroxysafflor yellow A (HSYA), salvianolic acid A (SAA), and salvianolic acid B (SAB) was effective for treatment of cerebral ischemia-reperfusion (I/R) injury in rats. However, the pharmacokinetic characteristics of this formula require further investigation. The present study investigated the pharmacokinetic differences between each component of in two formulas in cerebral I/R injury rats. The influencing factors may affect the compatibility of components were analyzed.

**Methods:**

Focal cerebral I/R was induced by middle cerebral artery occlusion (MCAO). Rats that underwent MCAO were randomly divided into two groups and administered treatments through the tail vein. Blood samples were collected at predetermined time points following administration. The concentrations of DSS, HSYA, SAB, and SAA in rat plasma were determined using HPLC-DAD, and the main pharmacokinetic parameters were calculated. Pharmacokinetic parameters were calculated using DAS 3.2.6 software and SPSS 23.0 statistical analysis software.

**Results:**

Our results showed that DSS, HSYA, SAB, and SAA in MCAO model rats had statistically significant differences in two formulas. For DSS and SAA, pharmacokinetic parameters with statistically significant differences including AUC_(0−t)_, AUMC_(0−t)_, MRT_(0−t)_, VRT_(0−t)_, t_1/2z_, V_z_, CL_z_, and C_max_ (*P* < 0.01). For HSYA, significant differences in the parameters including AUC_(0−t)_, AUMC_(0−t)_, MRT_(0−t)_, VRT_(0−t)_ (*P < *0.01), CL_z_ and C_max_ (*P < *0.05).

**Conclusion:**

The difference in pharmacokinetic parameters in response to each component may have been due to differences in the dosages of the components (HSYA, SAA, SAB) and the compatibility of components. Meanwhile, there were many influencing factors could affect the compatibility of components, such as the metabolism by CYP450 enzymes, plasma protein binding rates, and effects related to the blood-brain barrier (BBB). Moreover, our study provided new insights, such as choosing appropriate dosages of active components of traditional Chinese medicine (TCM) to aid in prevention and treatment of cerebral ischemic diseases. The method and results in this study could provide a foundation for future pharmacological studies of the active components in Danshen and Honghua.

## Introduction

Ischemic stroke is a common cerebrovascular disease (CVD) and the most common cause of death worldwide (Lozano et al., 2012). It is characterized by sudden loss of blood circulation to an area of the brain, resulting in a corresponding loss of neurologic function (Donnan et al., 2008). It’s the incidence of ischemic stroke is increasing with an aging population (Chen et al., 2017c). The global burden of ischemic strokes is nearly fourfold more common than hemorrhagic strokes (Kalaria et al., 2016). Cerebral ischemia-reperfusion (I/R) injury can occur during stroke treatment. This type of injury is of great concern because of the poor efficient medicine and clinical management.

**Figure 1:**
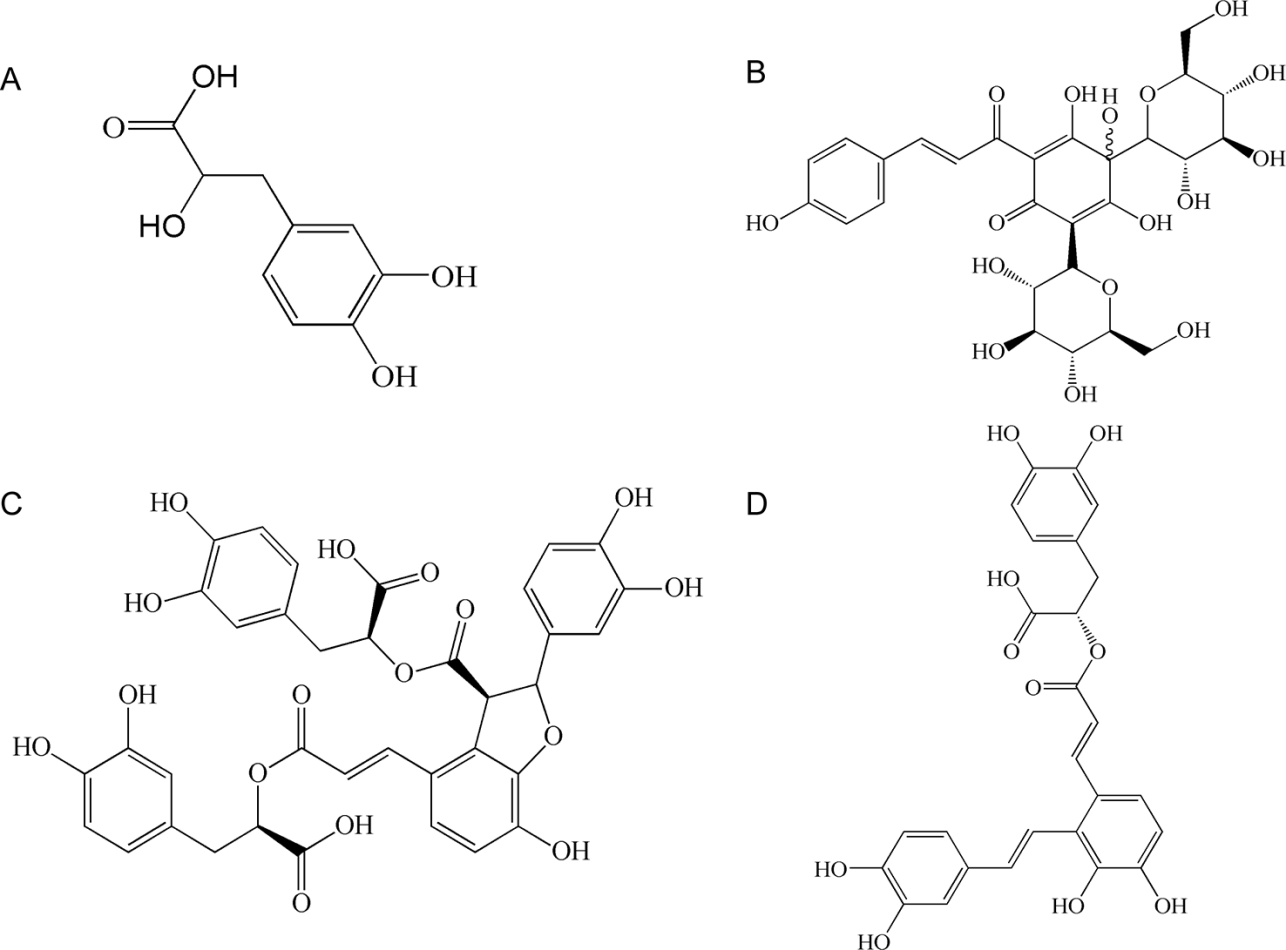
The chemical structure of four components: **(A)** danshensu (DSS); **(B)** hydroxysafflor yellow A (HSYA); **(C)** salvianolic acid B (SAB); **(D)** salvianolic acid A (SAA).

“*Salvia miltiorrhiza Bge.* (Danshen)- *Carthamus tinctorius L.* (Honghua)” is a known herb pair used in ancient traditional Chinese medicine (TCM) prescriptions for ischemic stroke (Li et al., 2013). This herb pair has been used historically in China and other countries in Asia to treat cardiovascular and CVDs (Qin et al., 2009; Liu et al., 2011; Chen et al., 2017b; Zhang et al., 2017b). Danshensu (DSS), salvianolic acid A (SAA), and salvianolic acid B (SAB) are the main hydrophilic components of Danshen (**Figure 1**) (Li et al., 2018c). Hydroxysafflor yellow A (HSYA) is a bioactive component of the dried flower of Honghua (**Figure 1**) (Hui et al., 2018). Studies have shown that DSS, SAA, SAB, and HSYA have therapeutic effects on cardiovascular and CVDs, through anti-inflammatory, anti-oxidative activities, etc (Fan et al., 2017; Feng et al., 2017; Xu et al., 2017; Sun et al., 2018).

Our previous study showed that DSS, HSYA, SAB, and SAA in combination protected against cerebral I/R injury in rats by inhibiting the response of endoplasmic reticulum (ER) stress and inflammation (Chen et al., 2018). The previous results showed that the protective effects of the combination group four (CG4) and six (CG6) on cerebral I/R were more significant in nine dosage-changing combination groups (Chen et al., 2018). Compared with the model group, CG4 (formula was as follows: 30 mg/kg DSS + 2.5 mg/kg SAA + 16 mg/kg SAB + 8 mg/kg HSYA) and CG6 (formula was as follows: 30 mg/kg DSS + 10 mg/kg SAA + 8 mg/kg SAB+ 4 mg/kg HSYA) treatment displayed that the neurological deficit scores were significantly reduced in cerebral I/R model rats (*P* < 0.05). In terms of ER stress, CG4 and CG6 treatment displayed that the mRNA expression of GRP-78 (*P* < 0.01) was significantly increased and the mRNA expression of CHOP was significantly decreased (*P* < 0.01). Meanwhile, in terms of inflammation, CG4 and CG6 treatment displayed that the protein expression of NF-κB p65 and the mRNA expressions of nuclear factor-kB (NF-κB), tumor necrosis factor-α (TNF-α), and interleukin-6 (IL-6) were significantly decreased in the cerebral cortex (*P* < 0.01).

However, the pharmacokinetic characteristics of these two groups (CG4 and CG6) have not been characterized. Therefore, the present study evaluated the pharmacokinetic differences of each component in two formulas (CG4 and CG6) in rats that underwent cerebral I/R injury. We also analyzed the potential factors which could affect the compatibility of these active components.

## Materials and Methods

### Chemicals and Reagents

Danshensu (DSS) (purity ≥98%, batch No. SZ201707038DSS), HSYA (purity ≥98%, Batch No. SZ201702005QA), SAB (purity ≥98%, Batch No. SZ201706003DB), and SAA (purity ≥98%, Batch No. SZ201706001DA) were purchased from Shizhou Biological Technology Co., Ltd (Nanjing, China) for use in plasma analysis. *p*-Hydroxybenzoic acid [internal standard (IS), purity ≥99%] was purchased from Guangfu Chemical Research Institute (Tianjin, China). Heparin sodium (Batch No. 2B010350) was purchased from Dingguochangsheng Biotechnology Co., Ltd (Beijing, China). HPLC grade methanol and acetonitrile were obtained from Tedia company. Pure water was supplied by a Millipore pure water system (Millipore, America).

### Experimental Animals

Adult male Sprague–Dawley (SD) rats, weighing 280 ± 20 g, were obtained from the animal experiment center of Zhejiang Chinese Medical University (Certification No. SCXK 2014-0001). The animals were housed in an environmentally controlled room (temperature: 25 ± 2°C, humidity: 45 ± 5%) for at least 5 days prior to experimental procedures. The rats were fasted overnight with free access to water prior to experiments. Animal welfare and experimental procedures were strictly in accordance with the Regulation for the Administration of Affairs Concerning Experimental Animals (State Science and Technology Commission, 1988) and approved by the Animal Subjects Review Board of Zhejiang Chinese Medical University.

### Focal Middle Cerebral Artery Occlusion

The MCAO model was implemented according to the method of Longa et al. (Longa et al., 1989). The rats were anesthetized with 10% chloral hydrate solution (0.3 ml/100 g) by intraperitoneal injection. An incision was made in the skin, and the right common carotid artery (CCA), the external carotid artery (ECA), and internal carotid artery (ICA) were isolated. The ECA and CCA were ligated, and a 0.26-mm polylysine-coated nylon monofilament was introduced into the right ICA through the CCA to occlude the middle cerebral artery (MCA) in the brain. After 1 hour of occlusion, the suture was withdrawn to allow reperfusion for 23 hours. A heating pad was used to maintain a core temperature of 37 ± 0.5°C during the surgery.

### Pharmacokinetic Study

Rats that underwent MCAO were randomly assigned to the following two groups (n = 5 per group): CG4 (30 mg/kg DSS + 8 mg/kg HSYA + 16 mg/kg SAB + 2.5 mg/kg SAA) and CG6 (30 mg/kg DSS + 4 mg/kg HSYA + 8 mg/kg SAB + 10 mg/kg SAA). The drugs were prepared by 0.9% saline. After reperfusion for 23 hours following 1 hours of cerebral ischemia, each group was administered the drug formulas *via* the tail vein.

After intravenous injection, 0.5 ml of blood was collected from the jaw vein after 2, 5, 10, 15, 30, 45, 60, 90, 120,150, 180, and 240 minutes respectively. In addition, 18 μl of heparin sodium was added as an anticoagulant. After centrifugation at 4,000 rpm for 12 minutes, plasma samples were transferred to clean tubes and stored at −20°C until analysis.

### Method Validation

Standard stock solutions of DSS, HSYA, SAB, and SAA were prepared in methanol at a concentration of 1 mg/ml. Six different concentrations of reference standard solution were prepared in 100 μl of blank rat plasma with appropriate volumes of the standard stock solution. The final concentrations in plasma were 1, 2, 8, 25, 50, and 100 μg/ml for DSS; 1, 2, 8, 15, 30, and 60 μg/ml for HSYA; 1, 2, 7.5, 30, 60, and 120 μg/ml for SAB; and 1, 2, 12.5, 50, 100, and 200 μg/ml for SAA.

*p*-Hydroxybenzoic acid was prepared as an IS in methanol at a concentration of 1 mg/ml solution. The IS solution was diluted tenfold (0.1 mg/ml) for experimental use.

These standard solutions were subjected to the entire analytical procedure to validate the linearity, accuracy, precision, recovery, and stability [quality control (QC)] of the method.

### Plasma Sample Preparation

The plasma samples (100 μl) were mixed with 10% phosphoric acid (5 μl), 10 μl of IS solution, and 300 μl of methanol. The mixture was vortexed for 30 seconds and centrifuged at 12,000 rpm for 10 minutes at 4°C. After centrifugation, the supernatant was transferred to another tube, and the extract was evaporated to dryness under a gentle stream of nitrogen. The residue was reconstituted with methanol:0.1% phosphoric acid (50:50, v/v), then centrifuged at 12,000 rpm for 10 minutes at 4°C. The supernatants were analyzed using HPLC after centrifugation.

### Instrumentation and Chromatographic Conditions

Analysis was performed on an Agilent 1200 series HPLC system (including G1311A quaternary gradient pump, G1316A column temperature box, G1315D diode array detector, G1322A on-line degasser, and chemical workstation). Chromatographic separation was achieved using an Eclipse XDB-C18 (5 μm, 4.6 mm × 250 mm) analytical column at maintained at 30℃. The mobile phases were acetonitrile (mobile phase A) and 0.1% phosphoric acid (mobile phase B). The gradient elution procedure was as follows: 0–17 minutes, 9%–33% A; 17–24 minutes, 33%–40% A; and 24–31 minutes, 40%–9% A. The flow rate was 1 ml/min. The detector was set to dual-wavelength detection at 280 nm for DSS, SAB, and SAA, and 403 nm for HSYA. The injection volume was 20 μl.

### Data Processing and Statistical Analysis

Pharmacokinetic parameters for DSS, HSYA, SAB, and SAA were calculated from the plasma concentration versus time data using Drug and Statistic Version 3.2.6 (DAS 3.2.6) software (the Mathematical Pharmacology Committee, Chinese Pharmacological Society, China). Experimental data and pharmacokinetic parameters were expressed as the mean ± standard deviation (SD). Variance analysis was using SPSS 23.0 statistical software.

## Results

### Specificity

**Figure 2:**
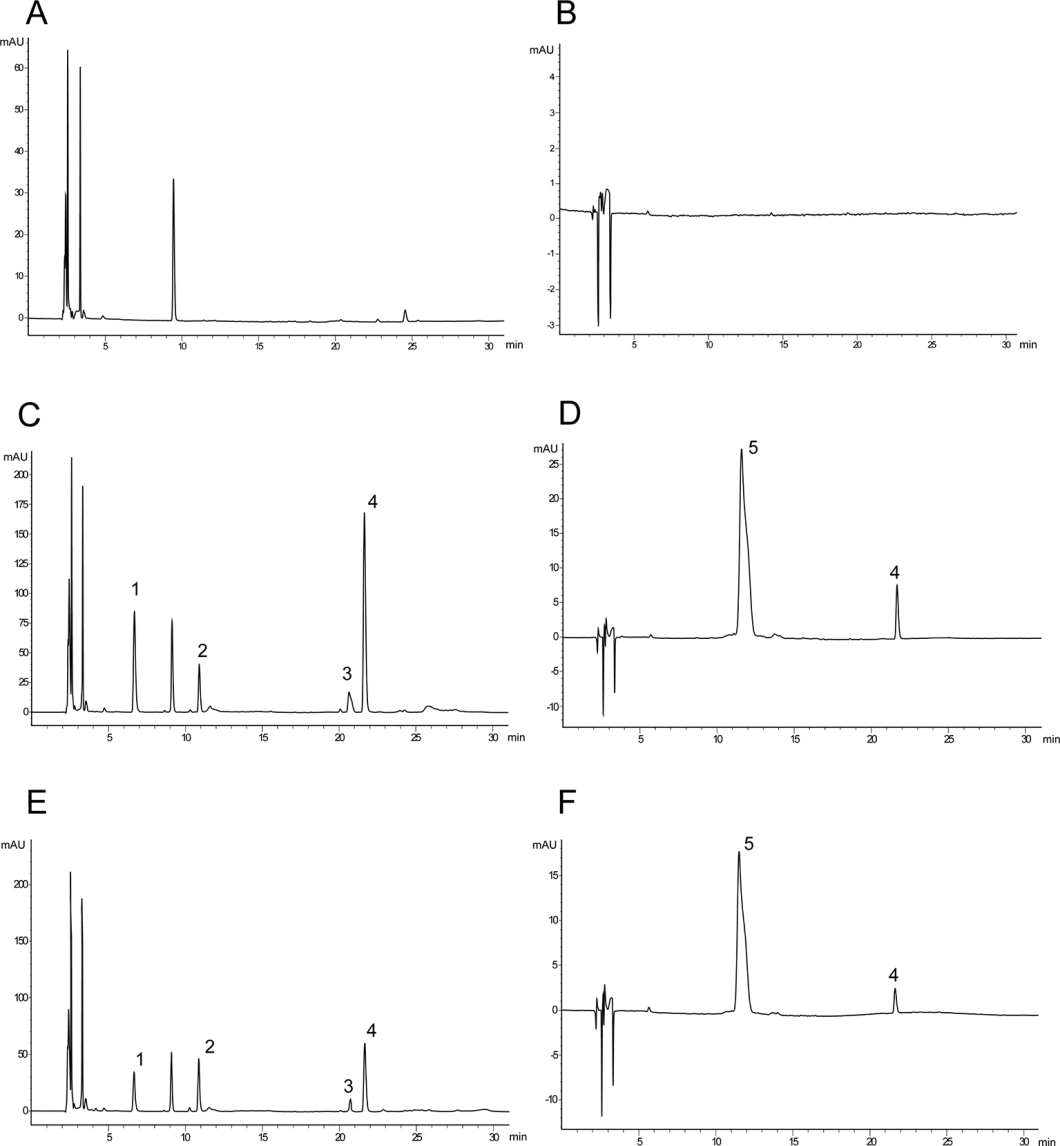
Typical chromatograms of rat plasma. **(A)** Blank serum sample (280 nm); **(B)** blank serum sample (403 nm); **(C)** blank serum sample spiked with danshensu (DSS), hydroxysafflor yellow A (HSYA), salvianolic acid B (SAB), salvianolic acid A (SAA), and internal standard (IS) (280 nm); **(D)** blank serum sample spiked with HSYA and *p*-hydroxybenzoic acid (403 nm); **(E)** after 10 minutes of administration (280 nm); **(F)** after 10 minutes of administration (403 nm) (1: DSS; 2: *p*-hydroxybenzoic acid (IS); 3: SAB; 4: SAA; 5: HSYA).

The chromatograms showed baseline separation of DSS, HSYA, SAB, SAA, and *p*-hydroxybenzoic acid without any interference from components of plasma (**Figure 2**).

### Investigation of Linearity and Lower Limit of Detection

All analytes were linear across the ranges evaluated. In **Table 1**, *Y* was the peak-area ratio of the analytes to the IS and *X* was the plasma concentration of the analytes. The lower limit of detection (LLOD) was defined as the level at which the signal to noise ratio was 3. The LLOD for DSS, HSYA, SAB, and SAA were 0.14, 0.09, 0.21, and 0.1 μg/ml, respectively.

**Table 1:** Linearity for the analysis of danshensu (DSS), hydroxysafflor yellow A (HSYA), salvianolic acid B (SAB), and salvianolic acid A (SAA) under standard solutions.

Analyte	Calibration curve	Correlation coefficient (*r*)	Linearity（mg/L）
DSS	*Y* = 0.0341*X* + 0.0506	0.9956	1~100
HSYA	*Y* = 0.0881*X* + 0.0472	0.9975	1~60
SAB	*Y* = 0.0113*X* + 0.0549	0.998	1~120
SAA	*Y* = 0.1226*X* + 0.0881	0.9994	1~200

### Accuracy and Precision

Intra-day precision was evaluated at five different times on the same day, and inter-day precision was evaluated on five different days in a week. The results were summarized in **Table 2**. The interday and intraday precisions values for DSS, HSYA, SAB, and SAA, expressed as percent relative standard deviations (%RSD), were less than 10% at each concentration. Accuracy, expressed as the percent relative error (%RE) was also less than 10% for each analyte at each concentration. These results indicated that the method was reliable and reproducible for biological sample analysis.

**Table 2:** Precisions and recoveries of each reference substance (*n* = 5).

Analyte	Concentration (mg/L)	Recovery (%, mean ± SD)	Intra-day		Inter-day	
			RSD (%)	RE (%)	RSD (%)	RE (%)
DSS	12.5	100.4 ± 0.1	0.9	–9.8	0.8	–6.9
	25	107.3 ± 0.1	0.4	1.9	0.4	–2.0
	50	109.3 ± 3.2	0.5	–3.3	0.4	0.4
HSYA	7.5	90.0 ± 0.1	0.9	–7.6	0.5	–4.0
	15	86.6 ± 0.2	0.4	–3.8	0.5	–6.5
	30	93.6 ± 0.8	0.5	–6.7	0.6	–3.3
SAB	15	128.3 ± 1.2	1.5	–6.9	5.4	8.1
	30	111.4 ± 0.8	0.5	7.7	4.3	–5.9
	60	67.5 ± 2.0	2.2	–8.2	1.6	–6.2
SAA	25	102.4 ± 0.1	0.4	–3.8	1.5	–3.7
	50	79.4 ± 0.3	0.5	–2.2	0.5	–5.3
	100	84.5 ± 2.1	0.6	–4.7	0.7	–2.6

### Recovery

Recovery is the measure of the ability to extract an analyte test samples. The recoveries of DSS, HSYA, SAB, and SAA from plasma are summarized in **Table 2**.

### Stability

Control solutions of DSS, HSYA, SAB, and SAA at three different concentrations (**Table 2**) were spiked into blank rat plasma. The drug-containing plasma samples were frozen at −20°C, thawed three times, then pretreated, and injected onto the HPLC. The stability of the method for each sample was expressed as the relative standard deviation (%RSD). The results were 0.363, 0.310, and 0.923% for DSS; 0.471, 0.420, and 0.533% for HSYA; 1.496, 1.497, and 3.479% for SAB; and 0.165, 0.219, and 0.874% for SAA. The %RSD values obtained in the stability study were less than 10%. The results showed that the samples were stable after repeated freeze-thaw cycles.

### Pharmacokinetics Study

**Figure 3:**
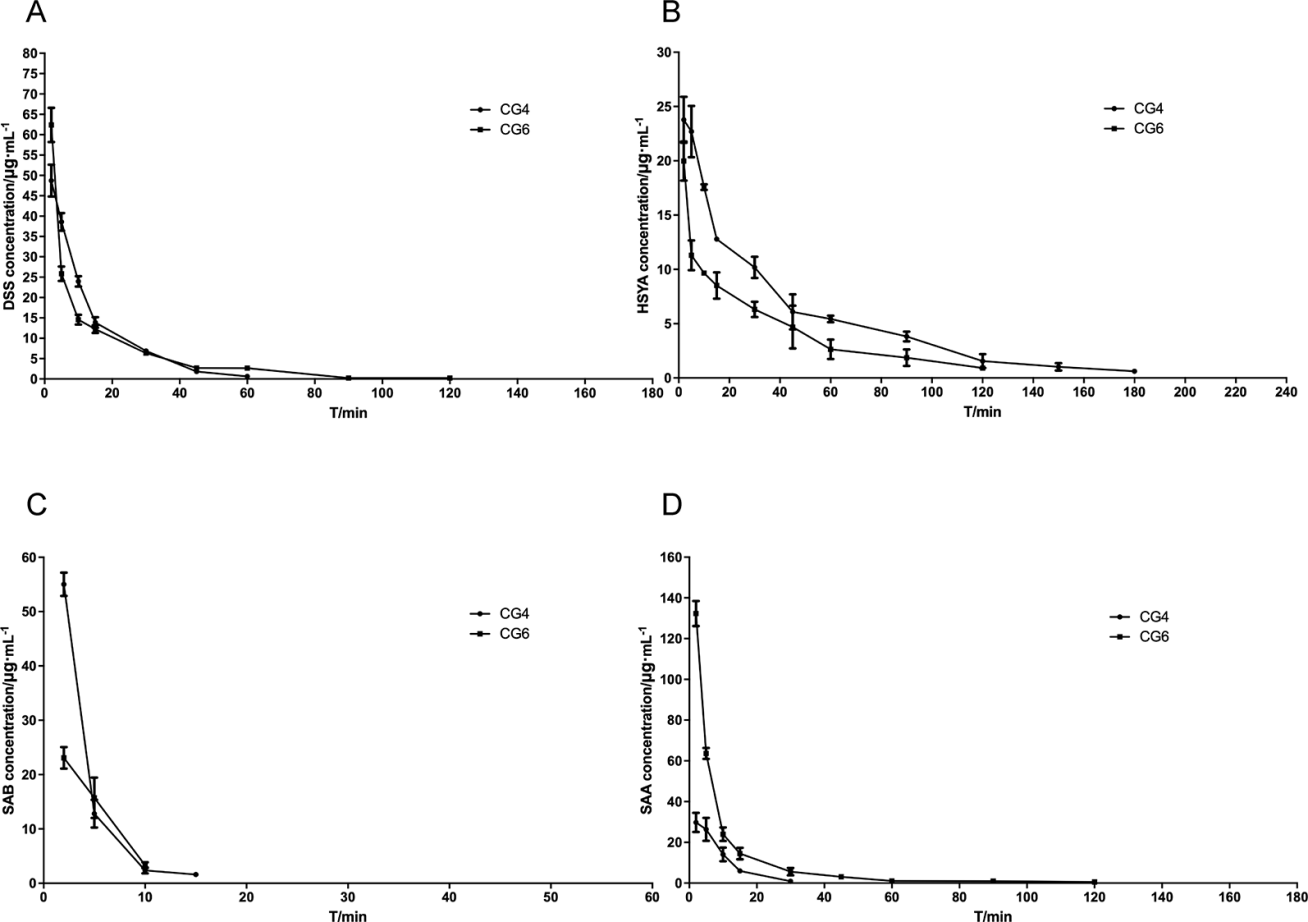
Blood concentrating-time of components. **(A)** danshensu (DSS); **(B)** hydroxysafflor yellow A (HSYA); **(C)** salvianolic acid B (SAB); **(D)** salvianolic acid A (SAA).

The plasma concentration-time curve in rats with MCAO is shown in **Figure 3**, and the main pharmacokinetic parameters from non-compartmental model analysis are summarized in **Table 3**.

**Table 3:** Pharmacokinetic parameters of danshensu (DSS), hydroxysafflor yellow A (HSYA), salvianolic acid B (SAB), and salvianolic acid A (SAA) in different groups (*n* = 5).

Parameters	Unit	DSS		HSYA		SAB		SAA	
		CG4	CG6	CG4	CG6	CG4	CG6	CG4	CG6
AUC_(0−t)_	mg/L*h	12.117 ± 0.075	13.164 ± 0.022**	16.206 ± 0.134	8.994 ± 0.159**	5.783 ± 0.27	/	5.816 ± 0.129	20.91 ± 0.048**
AUMC_(0−t)_	h*h*mg/L	2.479 ± 0.062	4.453 ± 0.017**	11.633 ± 0.139	5.068 ± 0.125**	0.196 ± 0.05	/	0.688 ± 0.026	4.155 ± 0.028**
MRT_(0−t)_	h	0.205 ± 0.019	0.338 ± 0.016**	0.718 ± 0.039	0.563 ± 0.081**	0.034 ± 0.031	/	0.118 ± 0.083	0.199 ± 0.029**
VRT_(0−t)_	h^2^	0.04 ± 0.016	0.211 ± 0.021**	0.456 ± 0.061	0.258 ± 0.076**	0.002 ± 0.001	/	0.009 ± 0.003	0.107 ± 0.015**
t_1/2z_	h	0.17 ± 0.025	0.376 ± 0.072**	0.66 ± 0.192	0.535 ± 0.281	0.03 ± 0.011	/	0.085 ± 0.059	0.504 ± 0.013**
V_z_	L/kg	0.599 ± 0.075	1.217 ± 0.015**	0.451 ± 0.12	0.318 ± 0.12	0.119 ± 0.043	/	0.052 ± 0.018	0.342 ± 0.057**
CL_z_	L/h/kg	2.444 ± 0.032	2.242 ± 0.019**	0.476 ± 0.11	0.412 ± 0.098*	2.76 ± 0.135	/	0.422 ± 0.016	0.47 ± 0.032**
C_max_	mg/L	48.721 ± 0.091	62.388 ± 0.12**	24.261 ± 0.137	19.978 ± 0.481*	55.031 ± 0.151	/	29.781 ± 0.047	132.347 ± 0.121**

Values are given as mean ± SD of five rats. *: *P* < 0.05; **: *P* < 0.01; compared with CG4.

After injecting the two combined medications groups separately, DSS and SAA induced statistically significant differences in AUC_(0−t)_, AUMC_(0−t)_, MRT_(0−t)_, VRT_(0−t)_, t_1/2z_, V_z_, CL_z_, and C_max_ (*P <*0.01). In addition, HSYA induced statistically significant differences in AUC_(0−t)_, AUMC_(0−t)_, MRT_(0−t)_, VRT_(0−t)_ (*P <*0.01), CL_z_, and C_max_ (*P <*0.05). Although the mechanisms are unclear, differences were observed *in vivo* following intravenous administration.

## Discussion

In this study, an HPLC-DAD method for simultaneous determination of DSS, HSYA, SAB, and SAA in MCAO model rats was established. Methanol was chosen as the protein-precipitating solvent during sample pretreatment to improve the peak shapes of the analytes. The reliability of this analytical method was confirmed by validating the specificity, linearity, precision, recovery, and stability of the method. The method was used to analyze the relationship between plasma concentration and time.

Cerebrovascular disease (CVD) is a leading cause of morbidity and mortality worldwide (Mozaffarian et al., 2016). Ischemic cerebral vascular disease (ICVD) is the predominant form of CVD (Wan et al., 2015). Ischemic stroke is a serious threat to human health and is the most frequent cause of permanent disability in adults worldwide (Lozano et al., 2012). The pathophysiological processes in ischemic stroke are diverse, and include inflammation, apoptosis, oxidative stress, intracellular calcium overload, and destruction of the blood brain barrier (BBB) (Tobin et al., 2014; Li et al., 2016; Li et al., 2018a).

TCM herbs and their components have been widely used as therapeutic agents in China since ancient times (Sun et al., 2015). Danshen and Honghua, a well-known “herb pair,” can stimulate blood circulation and dissipate blood stasis (Gao et al., 2016). The compositions of TCMs are very complex, and clinical effects often depend on composite effects of multiple components (Zhou et al., 2018). We previously showed that the combination of four herbal components in Danshen and Honghua protected against cerebral I/R injury in rats (Chen et al., 2018). Therefore, combinations of different classes of effective components with complementary mechanisms could result in improved therapeutic results.

Due to the severe symptoms and complications following strokes, the physiological status of patients can vary over time and between individuals. Pharmacokinetic studies to evaluate treatment of strokes are critical. This study was the first to investigate the effects of cerebral I/R on the pharmacokinetic compatibility for four herbal components. This was also the first study to compare the pharmacokinetic properties of these drugs in MCAO model rats. The pharmacokinetic parameters for DSS, HSYA, SAB, and SAA were significantly different in formulas of CG4 and CG6.

Compared with CG4, the pharmacokinetic parameters for DSS in CG6 showed that the exposure to DSS in plasma was significantly enhanced, the residence time was prolonged, and the clearance rate was reduced (*P* < 0.01). This result indicated that changes in the dosages of other components (HSYA, SAA, SAB) may have influenced the pharmacokinetics of DSS *in vivo* when the dosage of DSS was constant. Compared with CG4, the pharmacokinetic parameters for HSYA in CG6 showed that the absorption rate, the residence time, and the clearance rate were decreased. The opposite effect was observed for SAA. The results showed that increased SAA dosage resulted in increased AUC_(0−t)_, AUMC_(0−t)_, MRT_(0−t)_, VRT_(0−t)_, and C_max_ in CG6 compared to those in CG4 (**Table 3**). As shown in **Figure 3**, after administration, SAB was detected 2 minutes after administration. However, SAB was rapidly catabolized and was not detected 15 minutes after administration of CG6 formula. These results may have been due to lower sensitivity for, and lower dosages of, SAB (CG4: 16 mg/kg; CG6: 8 mg/kg). These results indicated that increasing or decreasing the dosage of a component impacted the pharmacokinetics of other components.

After reviewing the literature, we found that metabolic differences in the evaluated components may have been related to the compatibility of components. There are many influencing factors could affect the compatibility of components in addition to changes in the dosages of the components.

Cytochrome P450 (CYP450) enzymes are the main enzymes involved in human enzymatic metabolism (Tracy et al., 2016), and account for about 75% of drug metabolism in the human kidneys, liver, and intestines (Guengerich, 2008). Moreover, CYP450 enzymes are also key factors in drug interactions caused by the combination of TCM-TCM pairs and TCM-Western medicine pairs (Lu et al., 2015). Drug interactions that active or inhibit P450 enzymes can alter rates of P450-mediated metabolism (Tracy et al., 2016). A previous study showed that SAA can competitively inhibit CYP2C8 and partially inhibit CYP2J2 (Xu et al., 2018). DSS was shown to be a competitive inhibitor of CYP2C9 (Qiu et al., 2008). HSYA was shown to inhibit the activity of CYP1A2 and CYP2C11, and to increase the activity of CYP3A1 (Xu et al., 2014). Another report showed that injections that contain Danshen, or other formulations with high levels of tanshinones may lead to Danshen-drug interactions (Chen et al., 2017a). Therefore, if the two combination groups were to be administered separately, different pharmacokinetics may be observed due to altered rates of P450-mediated metabolism. These results agreed with our experimental results, which showed that the observed higher values of AUC_(0–t)_ and C_max_ in CG6 than CG4 possibly resulted from decreased elimination of DSS (**Table 3**). And the slower metabolism of DSS in CG6 may be due to the rates of CYP450-mediated metabolism, which could be altered by the interaction of components. And the CL_z_ of HSYA in CG6 was significantly lower than that in CG4 (**Table 3**). So, the metabolic rates of CYP450 enzymes is one of the important factors that may influence the compatibility of components and the pharmacokinetic processes of them in two formulas in addition to changes in the dosage of components.

Plasma protein binding rate is associated with drug-drug interactions, which can affect the absorption, distribution, metabolism, and excretion of drugs *in vivo* (Bohnert and Gan, 2013). According to previous reports, DSS had a low plasma protein binding rate (Zhang et al., 2017a), and HSYA, SAB, and SAA had a high plasma protein binding rate (Jing et al., 2010; Wang et al., 2011; Chen et al., 2017a). Thus, in the process of drug metabolism, the loss rate of SAA, SAB, and HSYA were high. In pharmacokinetics, the distribution is described by the parameter V, the apparent volume of distribution. And low plasma binding usually means an extensive tissue distribution (Oie, 1986). These results agreed with our experimental results which showed that, compared with CG4, the parameter V_z_ for DSS in CG6 showed the more extensive distribution, while the dosage of DSS was constant (*P* < 0.01). In addition, the parameter V_z_ for HSYA in CG6 showed no significant difference while the dosage of HSYA was decreased, compared with CG4 (*P* > 0.05). These results indicated that intravenous injection of a formula containing these four components may affect the compatibility of components *in vivo* due to their different plasma protein binding rates, leading to different pharmacokinetic process of each component in two formulas.

Previous studies have shown that administration of either DSS, HSYA, SAB, or SAA resulted in distinct mechanisms of protection against cerebral I/R damage, such reduction of inflammation and oxidative stress (Sun et al., 2010; Fan et al., 2017; Xu et al., 2017). The BBB is a dynamic interface between the blood and the brain parenchyma (Sifat et al., 2017; Jiang et al., 2018). The ability of a drug to cross the BBB is a key factor in therapeutic efficacy. The disruption of the BBB by I/R has been shown to contribute to increased levels of SAA in the brains of I/R model rats (Feng et al., 2017). When damage occurs, SAA can reach the brain through the BBB and induce therapeutic effects in cerebral I/R model rats. In addition, studies have shown that HSYA can cross the BBB, resulting in downregulation of 12/15-lipoxygenase (12/15-LOX) and its metabolic products (Sun et al., 2012), and attenuation of occludin, claudin-5, and ZO-1 expressions, resulting in BBB permeability and improvement of tight junctions in MCAO mice (Lv and Fu, 2018). Meanwhile, DSS can readily permeate the BBB when normal rats were orally administered of Danshen extract (Zhang et al., 2011). Another study showed that SAB distributed rapidly to blood-rich tissues, such as the kidney (Li et al., 2018b). These findings indicated the pharmacokinetic differences of these four components in two formulas may result from the different effects of the components on the BBB.

## Conclusion

In our study, an HPLC-DAD method for simultaneous determination of DSS, HSYA, SAB, and SAA in MCAO model rats was established. Comparison of the pharmacokinetic parameters of the four herbal components in different formulas in MCAO model rats showed that difference in pharmacokinetic parameters may have been related to the changes in dosages of the components (HSYA, SAA, and SAB) and the compatibility of components. Meanwhile, there were many influencing factors could affect the compatibility of components, such as the metabolic rates of CYP450 enzymes, plasma protein binding rates and the effects of the components on the BBB. Furthermore, this study may provide guidance for evaluation of pharmacokinetic parameters and pharmacological effects of active components of TCMs in pathological states.

## Data Availability Statement

The datasets generated for this study are available on request to the corresponding authors.

## Ethics Statement

Animal welfare and experimental procedures were strictly in accordance with the Regulation for the Administration of Affairs Concerning Experimental Animals (State Science and Technology Commission, 1988) and approved by the Animal Subjects Review Board of Zhejiang Chinese Medical University.

## Author Contributions

XZ, LY, HW, and JY participated in designing experiments, carried out the experiments in this study, prepared the first draft, and revising of this manuscript. YC performed the drug administration and blood sampling. YW participated in data analysis. All authors contributed to manuscript revision, read and approved the final manuscript.

## Funding

This research was financially supported by the Natural Science Foundation of Zhejiang Province (No. LZ17H270001) and the National Natural Science Foundation of China (No. 81874366, 81803992, 81873226).

## Conflict of Interest

The authors declare that the research was conducted in the absence of any commercial or financial relationships that could be construed as a potential conflict of interest.
